# Web-Based Platform for Systematic Reviews and Meta-Analyses of Traditional Chinese Medicine: Platform Development Study

**DOI:** 10.2196/49328

**Published:** 2024-11-22

**Authors:** Weiqiang Zhou, Dongliang Liu, Zhaoxu Yi, Yang Lei, Zhenming Zhang, Yu Deng, Ying Tan

**Affiliations:** 1 Hunan Academy of Chinese Medicine, 142 Yuehua Road, Yuelu District, Changsha, 410013, China, 86 73188855204

**Keywords:** evidence-based medicine, information science, medical librarian, web development, web design, meta-analysis, traditional Chinese medicine, systematic review, review methodology, Chinese medicine, traditional medicine

## Abstract

**Background:**

There are many problems associated with systematic reviews of traditional Chinese medicine (TCM), such as considering “integrated traditional Chinese and Western medicine” or treatment methods as intervention measures without considering the differences in drug use, disregarding dosage and courses of treatment, disregarding interindividual differences in control groups, etc. Classifying a large number of heterogeneous intervention measures into the same measure is easy but results in inaccurate results. In April 2023, Cochrane launched RevMan Web to digitalize systematic reviews and meta-analyses. We believe that this web-based working model helps solve the abovementioned problems.

**Objective:**

This study aims to (1) develop a web-based platform that is more suitable for systematic review and meta-analysis of TCM and (2) explore the characteristics and future development directions of this work through the testing of digital workflow.

**Methods:**

We developed TCMeta (Traditional Chinese Medicine Meta-analysis)—a platform focused on systematic reviews of TCM types. All systematic review–related work can be completed on the web, including creating topics, writing protocols, arranging personnel, obtaining literature, screening literature, inputting and analyzing data, and designing illustrations. The platform was developed using the latest internet technology and can be continuously modified and updated based on user feedback. When screening the literature on the platform, in addition to the traditional manual screening mode, the platform also creatively provides a query mode where users input keywords and click on *Search* to find literature with the same characteristics; this better reflects the objectivity of the screening with higher efficiency. Productivity can be improved by analyzing data and generating graphs digitally.

**Results:**

We used some test data in TCMeta to simulate data processing in a systematic review. In the literature screening stage, researchers could rapidly screen 19 sources of literature from among multiple sources with the manual screening mode. This traditional method could result in bias due to differences in the researchers’ cognitive levels. The query mode is much more complex and involves inputting of data regarding drug compatibility, dosage, syndrome type, etc; different query methods can yield very different results, thus increasing the stringency of screening. We integrated data analysis tools on the platform and used third-party software to generate graphs.

**Conclusions:**

TCMeta has shown great potential in improving the quality of systematic reviews of TCM types in simulation tests. Several indicators show that this web-based mode of working is superior to the traditional way. Future research is required to focus on validating and refining the performance of TCMeta, emphasizing the ability to handle complex data. The system has good scalability and adaptability, and it has the potential to have a positive impact on the field of evidence-based medicine.

## Introduction

### Background

RevMan [1], Stata [2], SPSS [3], and R [4] are frequently used software or tools for systematic reviews, mainly running on the computer desktop.

RevMan is Cochrane’s bespoke software for writing Cochrane Reviews. RevMan Web [5] is now available, on a subscription basis, for generating non-Cochrane reviews or for other evidence synthesis work [6] and is expected to replace RevMan 5. As of April 25, 2023, RevMan 5 is no longer recommended for download and use as a Cochrane editor and author [7]. Internet-based tools are currently replacing traditional desktop approaches.

Many other internet tools can perform meta-analyses. For example, MetaGenyo [8] is a useful tool for performing comprehensive genetic association meta-analyses [9]. MetaInsight [10] is an interactional web-based tool that uses R-shiny and netmeta to analyze, query, and visualize web-based meta-analyses. MetaDTA [11] addresses graphical enhancements to summary receiver operating characteristic curve maps to facilitate analysis and reporting of meta-analyses of data regarding the accuracy of diagnostic tests. PythonMeta [12] implements web-based generation of forest and funnel plots. Qiao et al [13] developed an evidence-based medical assistant platform that is mainly used to quantify and convert indicators.

Evidence-based traditional Chinese medicine (TCM) also has its own characteristics and needs. Dong and Liu [14] at the Centre for Evidence-Based Chinese Medicine at Beijing University of Chinese Medicine proposed some distinctive methodologies in response to the phenomenon of “four more” and “four less” in the development of evidence-based TCM, such as evaluating increases or decreases in drug ingredients when assessing efficacy to determine the optimal TCM treatment plan. They believe that it is also necessary to regulate the search terms [15]. Wang and Huang [16] and others from the China Academy of Chinese Medical Sciences believe that it is necessary to establish the China Center for Evidence Based Traditional Chinese Medicine (CCEBTCM). To carry out high-quality evidence-based research on the premise of respecting the objective laws of TCM, data can be collected and shared through the internet to establish a database of evidence-based TCM that can be systematically retrieved and invoked. Ji et al [17] from the Evidence Based Medicine Center at Tianjin University of Traditional Chinese Medicine developed the SMD-TCM (systematic review/meta-analysis database of traditional Chinese medicine) platform [18], which focuses on the reevaluation of systematic reviews and meta-analyses and provides efficient platform services for evidence translation research.

Studies on these platforms do not involve the management of processes in systematic reviews of TCM types. However, systematic reviews of TCM still face numerous quality issues that differ from those in Western medicine. Meta-analysis of clinical trial literature with varying dosage forms, dosages, usage, treatment courses, and controls may result in significant evaluation bias. A simple meta-analysis of TCM or a combination of traditional Chinese and Western medicine in the treatment of a disease is susceptible to logical errors due to overly broad intervention measures.

We believe that if the research process of a systematic review or meta-analysis is publicly available and can be reviewed or corrected, and detailed data are available, the quality of the results may be higher.

Attempts should be made to digitalize the research process of systematic reviews, so that the user’s research process can be viewed by the third party, which will help standardize the research process, find problems, and correct errors. The release of RevMan Web further indicates that conducting systematic review research on the web will be the trend in the future.

### Objective

In response to these considerations, the primary objective of this study is to propose TCMeta, which stands for “Traditional Chinese Medicine Meta-analysis,” an internet-based platform for systematic review of research more suitable for the characteristics of TCM. Users can set topics, form teams, import and screen literature, generate analytical illustrations, and conduct systematic reviews and meta-analyses. TCMeta was originally published on the internet in 2022 and is available on the web [19].

Another objective of this study is to simulate the production of a systematic review and perform a meta-analysis using this platform. Through the introduction of the entire workflow, this paper discusses the problems that need to be understood and considered in web-based work, elucidates the advantages of web-based work, and discusses the points that need to be improved in future work.

## Methods

### Overview

Based on the rule-oriented design concept, TCMeta designs in accordance with the standard process of a systematic review and the specific needs of TCM types and combines existing third-party internet-based tools to allow users to complete the entire business process on the web.

### Functional Architecture of TCMeta

**Figure 1.**
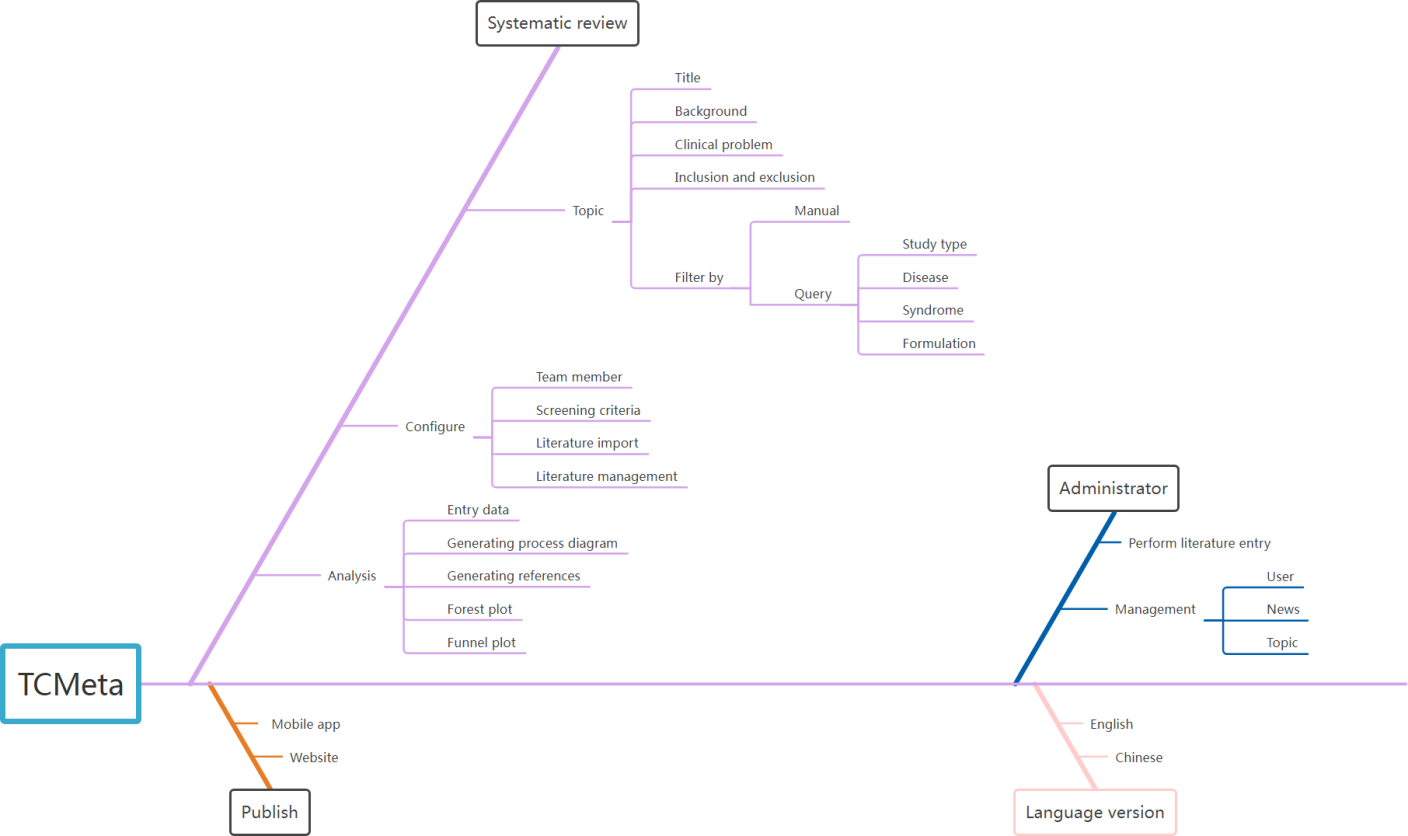
Functional architecture of TCMeta (Traditional Chinese Medicine Meta-analysis).

Figure 1 shows the functional architecture of TCMeta. As shown in the figure, TCMeta has the following key modules and features:

1. TCMeta has released a website and mobile app for public use and provides the function of menu switching between Chinese and English, which is convenient for users with different native languages.
2. The administrator is responsible for import and maintenance of the literature. Users can query and import literature indexes from external databases according to their needs, and the administrator ensures the uniqueness and health of the data in the database to avoid data duplication caused by multiple users importing the same literature. Administrators maintain and manage users and public information and can also professional suggestions for users’ topic selection.
3. Research usually involves 3 steps: topic selection, configuration, and analysis. The topic selection stage involves inputting research-related information including background description, clinical problems, inclusion and exclusion criteria, and research methods. Configuration includes the arrangement of researchers, importing and screening the literature, and determining which literature is selected. In the analysis phase, illustrations were generated on the web after inputting the relevant data to export.

### Application Development

TCMeta is developed based on Asp.NET 6.0 [20], uni-app [21] and Vue.js [22], including a website, a web-based application programming interface (API), and a mobile app. The front- and backends are separated and cross-platform operation is supported. uni-app is a front-end framework for developing cross-platform applications using Vue.js. Developers write a set of code that can be published on iOS, Android, the Web (responsive), and various small programs, fast apps, and other platforms. uni-app has strong competitive advantages in 6 key indicators: number of cross-ends, expansion capacity, performance experience, surrounding ecology, learning cost, and development cost. The backend uses Asp.Net 6.0, using technologies including AutoMapper [23], Asp.NET Core API, Newtonsoft Json.NET [24], MYSQL [25], and so on ().

### Web-Based Literature Screening

The user uses both manual and query modes to accomplish web-based literature selection.

The manual model is the traditional way of working in a systematic review. Users assign tasks in the form of a working group, and the group leader determines the theme and literature screening criteria, etc, and the group members import the literature and complete the screening independently. The data are mainly derived from third-party literature databases, such as PubMed [26] and Wanfang [27]. After searching and exporting the required documents, they were imported into this platform. After literature screening, the data were analyzed.

In query mode, the text of the imported literature is extracted. Users refer to the original literature, input the treatment plan, including treatment means, inclusion criteria, etc, and rapidly look for similar studies, or divide the search results into different subgroups. Although standard practice emphasizes the need for 2 researchers to complete the same work independently to reduce errors, omissions, or bias of the work, the quality of the work is inevitably affected by the cognitive level and subjective consciousness of the researchers. The query mode is based on objective data for easy checking and proofreading. In particular, there are numerous self-written prescriptions in clinical research involving TCM, and the query mode can help quickly find the same studies among them. In the query mode, the composition of self-designed medicines such as TCM decoctions can be separated, and the literature with the same or similar prescriptions can be found based on the necessary ingredients of a decoction. The composition of acupuncture points in acupuncture schemes can be divided, and the same or similar acupuncture schemes can be found by looking up combinations of acupuncture points. After inputting the text, the treatment schemes for the treatment and control groups in all sources of literature can be exported, including medication mode, drug name or type, drug ingredients, etc, so that users can quickly find the same treatment plan.

### Web-Based Data Analysis

After the user screens the literature on the web, a literature screening flowchart is generated, and the forest and funnel plots are further generated. The system will record the user’s literature screening process and automatically generate a flowchart. The flowchart can also be edited and refined by the user. Users can input relevant data on the web for the final included literature. The platform integrates third-party plug-ins, and forest and funnel plots can be generated on the web based on the data. Users can also export the included literature as an XLS file to generate bias analysis plots, forest plots, and funnel plots on third-party internet platforms.

### Ethical Considerations

In this study, we introduced the process and method of conducting evidence-based research on TCM on the web, using data mainly derived from the published open literature. No human participants or animals were directly involved in this study, and no additional risks or discomfort were incurred. Therefore, no ethical approval was required for this study.

## Results

### General Literature Screening

Here, we present a systematic review and meta-analysis of TCM on the web, entitled “The Therapeutic Effect of Zhitongrushen Decoction on Anal Sinusitis.” To enhance visual representation in funnel and forest plots, we refrain from strict evaluation of heterogeneity, literature quality, etc. Therefore, the analytical conclusions should not be applied directly to clinical practice.

The first approach to literature screening is the manual mode, wherein 2 individuals independently conduct the screening process. Upon registration and log-in, user A can create a new topic under the “My Topic” menu, inputting details such as the title, background information, clinical problem statement, inclusion and exclusion criteria, and the screening methodology. At this juncture, user A automatically assumes the role of team leader for this particular topic. User A can access the “Home/ProjectManage/Configuration” items and select the “Evaluate users, Criteria, Import, Management” option for further action. Upon registration, user B can provide their email ID to user A, who may then add user B as a group member in the “Evaluate users” feature. If user A prefers not to perform literature screening tasks, they may include user C as a member.

In this example, users B and C are designated as group members who also serve as literature screeners. Upon logging into the system, these 2 members comprehended information regarding the topic and conducted a search for relevant literature in the database on the basis of selection requirements. By using the “Document Import” function, group members can import information regarding the title of Chinese- or English-language papers from Wanfang and PubMed databases. User B conducts a literature search on Wanfang using the selected topic and search sentence pattern. Relevant literature is then selected in NoteFirst format, saved, and imported into this system. User C conducts a literature search on PubMed, selects relevant papers, and clicks the “Save” button. The “Format” of “Save citations to file” is set to CSV before saving. User C then navigates to “Home/ProjectManage/Configuration/Import/Add” and adds the literature by selecting “PubMed” as the data source and entering search keywords in the “Search term.” Finally, user C uploads the saved CSV file into the system. Naturally, users B and C may replicate each other’s literature search efforts to maximize the comprehensiveness of their research.

This platform enables the administrator review mechanism to ensure the uniqueness of the library documents. After logging in, the system administrator will see the literature import entries of users B and C. After checking the data, the system administrator will perform the database operation. For journal articles, the platform judges whether they have been stored in the library based on the title, author, publication date, journal, DOI, and other information. Every literature import operation of the user involves adding literature data to this database, as if building a new PubMed platform. To protect the author’s copyright, the platform does not save the full text of the paper.

After conducting the search, 19 relevant literature sources were curated for this topic. Within the list of papers, users can access the original text via an external link by simply clicking on it. This is made possible through URL rule implementation, whereby the web page jump address is set as “https://xueshu.baidu.com/s?wd=(paper title),” allowing for seamless navigation to the location of the desired paper in Baidu and subsequent access to its corresponding original source. Upon reviewing the source material, the user is able to specify exclusion criteria and proceed with literature selection in a systematic manner. Group members operate independently without mutual influence during screening. Using the web-based review function, users can conduct comprehensive literature reviews without necessitating paper downloads.

The team leader and members can click on the menu “Home/ProjectManage/Configuration/Criteria/Add” and input the document filtering rules. The example below lists 3 rules.

**Figure 2.**
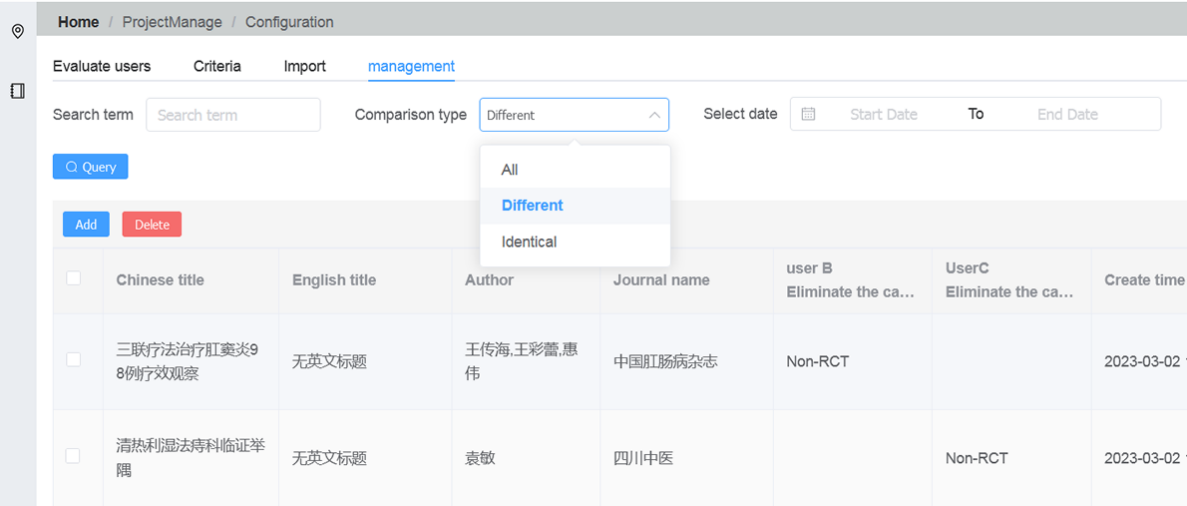
The differences in literature screening between 2 members were compared.

This platform offers an automatic comparison function. Once users complete the screening, group leaders can activate the “Look at each other” feature, allowing members to query differences and contrasts under “Home/ProjectManage/Configuration/Management/Comparison type.” After negotiation, filtering results are modified for complete consistency (Figure 2).

### Literature Screening Conducted in the Query Mode

The second approach to literature screening is through the query mode.

**Figure 3.**
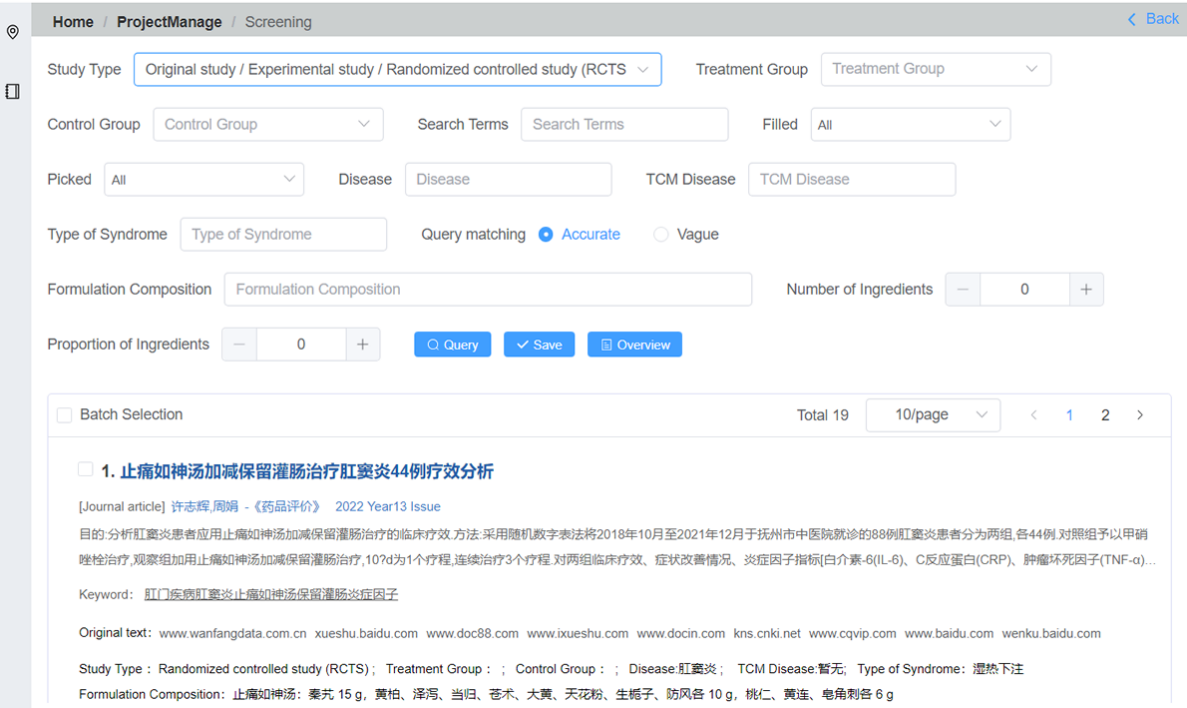
The primary interface for query mode.

Users input literature data into a preset table and can rapidly retrieve similar literature through search functions. Within the “Home/ProjectManage/Screening” menu, users first define the study type as a randomized controlled trial (RCT) or another type before inputting disease names according to Western medicine, disease names based on TCM, symptoms, syndromes, and TCM treatments. Then, the treatment methods used in both the treatment and control groups (such as sitz bath, fumigation, coating, cupping, massage, oral administration, acupuncture, and intravenous infusion) are documented, the composition of medicinal materials used in oral decoctions are elucidated, and acupuncture points targeted during treatments are specified. Additionally, names of drugs administered during treatments along with their specific contents are provided. If a user opts to divide TCM prescriptions, such information will be recorded within the detailed table (Figure 3).

The query mode is particularly suitable for TCM research and studies involving complex literature content. Users can create 2 versions of the same topic in manual and query modes, input data, compare literature, and divide subgroups using the query mode. Based on the results obtained from the query mode, users can screen literature in the manual mode to adhere to objective data as much as possible and reduce errors in subjective judgment.

After perusing the primary text, the researchers meticulously recorded each key element in separate tables. The recording process encompassed various aspects such as study type, abstract, objectives, diseases, clinical symptoms, and syndromes for both comparison groups with different treatments as depicted. Additionally, the prescription composition was analyzed to identify drug components.

After compiling the content of 19 articles, a search was conducted with the study type set to “Original Study/Experimental Study/Randomized controlled study (RCTs),” yielding 11 RCTs. The formulation composition of the Zhitongrushen decoction consists of 10 Chinese medicinal herbs including “tail of Angelica sinensis (Danggui Wei)” and “Rhubarb after steaming (Shu Dahuang).” These 10 herbs were entered as keywords separated by spaces in the textbox for multiword search.

The “Query matching/Accurate” option defines the degree of keyword matching as “same.” When searching “Angelica sinensis (Danggui),” the search results would not match those obtained with “tail of Angelica sinensis.” The “Query matching/Vague” option is defined as “inclusive,” allowing for the results obtained through a search of “Rhubarb (Dahuang)” to match those of both “Rhubarb” and “Rhubarb after steaming.” The “Number of Ingredients” option indicates how many keywords will be searched. The “Proportion of Ingredients” option indicates the proportion of all keywords in the target literature.

**Figure 4.**
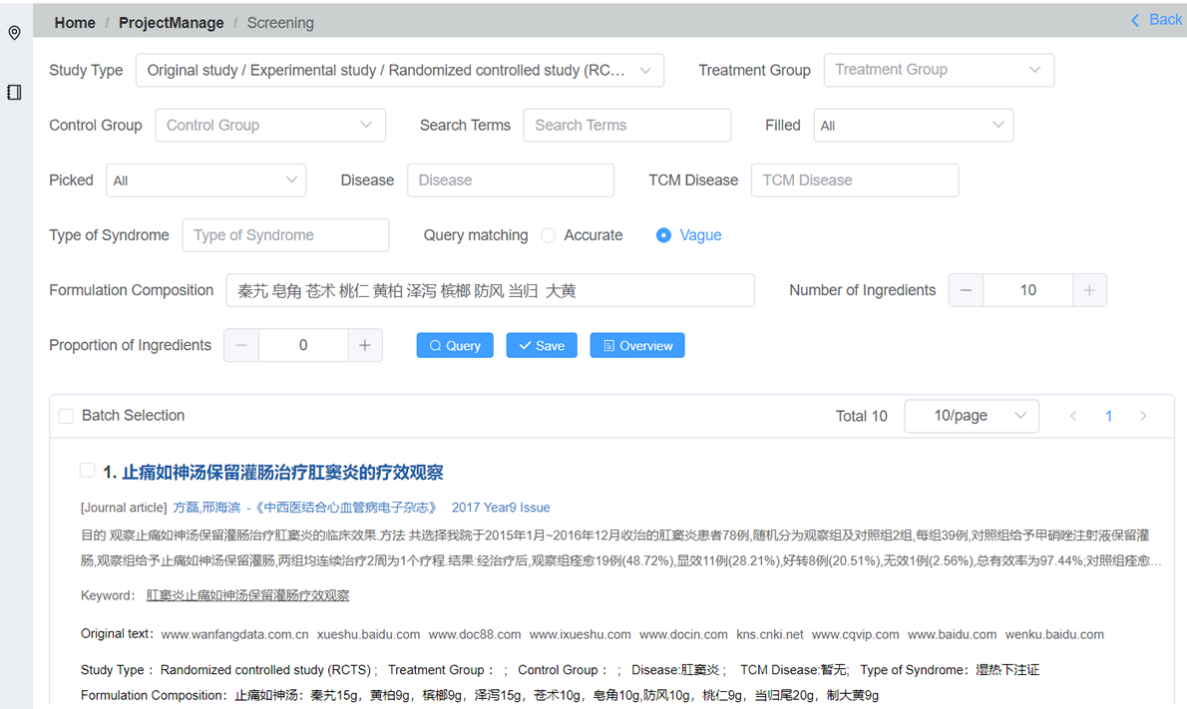
Use of query matching, number of ingredients, proportion of ingredients, and other options to find partially or exactly the same prescription.

In this example, “Accurate” was selected as the search option, the “Number of Ingredients” was set to 10, the “Proportion of Ingredients” was inputted as 100%, and upon searching, only 2 out of the 11 articles matched all keywords in terms of number and name in their prescriptions. Subsequently, “tail of Angelica sinensis” was revised to “Angelica sinensis” and “Rhubarb” after “steaming” was changed to “Rhubarb,” resulting in zero search results. Thereafter, the “Vague” option was chosen and another search was conducted, yielding 8 results. By iteratively adjusting the parameters during searching, users can gain insights into similarities and differences among prescriptions and generate a list of sources of literature with similar prescriptions (Figure 4).

By clicking on “Overview,” the user can access a comprehensive list of all entered data, which can be conveniently exported as an XLS file. After completing work in the query mode, the user can clearly understand the situation for all sources of literature and then return to the manual mode to complete the literature screening.

### Data Processing

**Figure 5.**
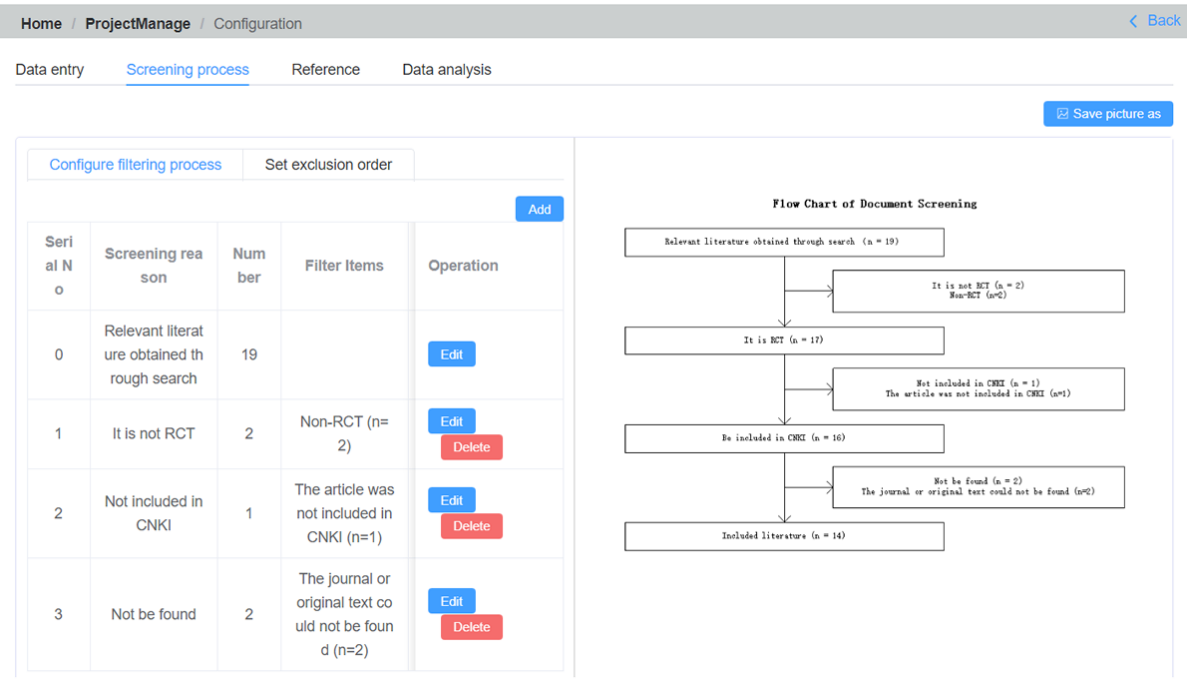
Flowchart for literature screening automatically generated by the program.

Data processing involves 4 steps: data entry, screening process, references, and data analysis. To input the selected literature data into the system, users can click on the “Data entry” option to access a list of chosen literature sources. They will then read through the original text and enter relevant information such as data type (continuous or binary variables), researcher name, publication year, experimental group details, and control group specifications. Upon clicking the “Screening process” button, the system will automatically display the previously executed “literature screening” exclusion process, detailing the number of documents initially searched, reasons for document removal, and a real-time flowchart that users can download (Figure 5). Users can modify descriptions, sort for filtering purposes, and refresh flowcharts in real time. However, to reflect the authenticity of the literature screening process, the number of articles screened under each rule cannot be modified here.

**Figure 6.**
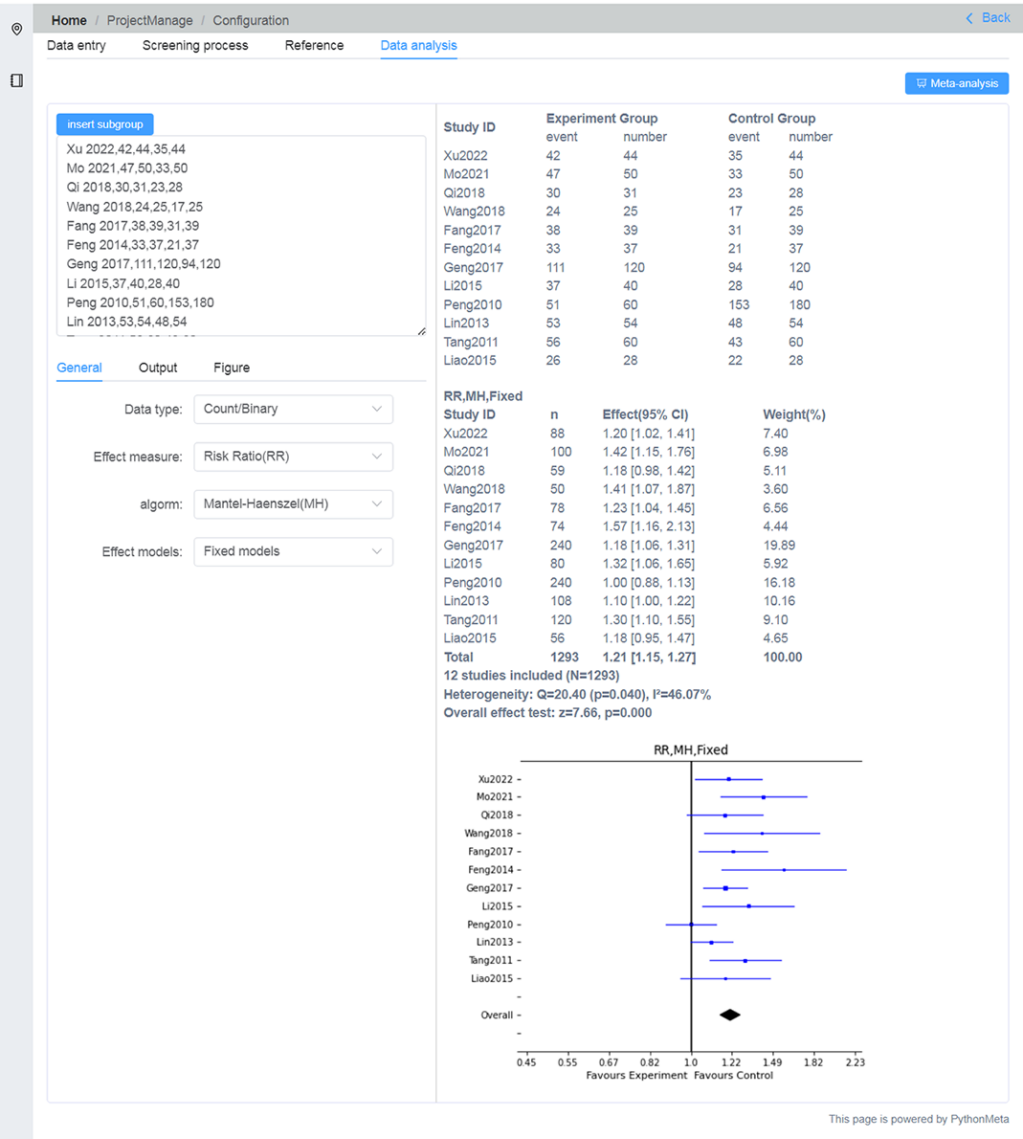
The forest plot was generated on the web via PythonMeta.

The “Reference” option presents a literature citation list, which can be easily copied or exported as an XLS file for use in the paper. The “Data analysis” option automatically generates a list of data based on the information entered in “Data entry.” In this case, we used PythonMeta [12] to produce web-based forest and funnel plots (Figure 6). More example operation diagrams are presented in Multimedia Appendix 2.

## Discussion

### Principal Results

This paper discusses how to develop an internet platform called TCMeta for evidence-based Chinese medicine research. In the abovementioned example, the web-based working model is introduced, the research process is reshaped, some methods and means are added, subgroups are more finely segmented, and heterogeneity in interventions is excluded to reduce bias and thus improve the quality of the research.

The application of some internet tools or platforms related to TCM has promoted the internationalization of research on TCM, such as BATMAN-TCM [28], an in silico analysis tool for molecular mechanisms in TCM; TCM-Mesh [29] for network pharmacology analysis; and TCMSP [30], a system pharmacology database for drug discovery from herbs. The convenience of the internet is obvious. After the operating system is upgraded or replaced, the used desktop software [31] may no longer be used, and the generated data cannot be easily exported and reused. Compared with desktop software [32], internet-based meta-analysis is faster and more convenient.

We are not only digitalizing our workplaces but also incorporating processes and methods to use them. Among the published articles on network meta-analysis in the field of TCM, most of them with the highest citation rate were methodological articles, including the introduction of methodological theory and application software [33], which indicates that the choice of methods was very important. We could organize the research work of systematic reviews on TCMeta, complete the literature screening, and output the data. Third-party tools for data analysis are displayed in TCMeta, which makes it convenient for researchers to choose and use according to their own needs. The platform also integrates and invokes open-source tools.

Traditional research methods of TCM focus on macroscopical and holistic aspects, while system review emphasizes on quantification and concreteness, which is contradictory. In this study, we quantify interventions, focus on handling of details, protect the objectivity of data, and attempt to exclude the interference caused by subjective feelings. In the field of TCM evidence–based research, web-based work can bring more benefits than offline work (Table 1).

**Table 1.** Advantages of working on the web compared to working offline.

Advantage	A cause or manifestation
Facilitate work	No matter the time or place
Work process can be checked	The operation of each link, including the change and processing of data, can be traced
Data reuse	The excluded documents or data are still in the platform and may be suitable for other types or topics of research
Data cannot be easily tampered with, much less falsified	The source and details of the data are public and searchable
Facilitate communication and collaboration among members	Know the current status of each other’s work
Gain inspiration	Some of the excluded documents may have some homogeneity and may be excellent material for other topics

### Strengths and Implications

Process determines success or failure, and details affect quality. Compared to other platforms related to systematic reviews, TCMeta has unique advantages and innovations, which could be easily overlooked, because it focuses on the field of TCM and developing workflows. Specifically, some argue that TCM is less suitable for producing and publishing systematic reviews, and some argue that data analysis is more important than data collation, thus neglecting the resolution of issues in TCM-type systematic reviews through process reengineering. We are focused on that, and we are already seeing initial results.

### Limitations and Future Work

The platform offers menus in both Chinese and English, with Wanfang and PubMed serving as the primary sources of literature. In future iterations, additional language options should be included to attract a wider range of users interested in evidence-based research on TCM. The expansion of sources for importing bulk literature should be considered to facilitate the acquisition of literature from databases in various countries. It is crucial to facilitate cross-border and cross-lingual exchanges and collaborations among users, as well as to promote the establishment and implementation of international evidence-based research projects on TCM. This platform will primarily focus on literature screening and management, while using third-party APIs for generating forest and funnel plots. We should further enhance our capabilities in web-based analysis and data visualization in subsequent releases.

### Conclusions

A variety of technologies and methods are needed to develop an internet-based platform suitable for evidence-based research on TCM. Some automatic processing methods are added to the workflow, such as identifying whether the imported literature is repeated, comparing the differences between the 2 members in literature screening, and automatically generating flowcharts according to the literature screening steps. The program design of data analysis is the difficulty of the work. In addition to innovation, it also involves the mutual invocation of different computer programs and services. With the advancement and development of technology, it is expected that more third-party–related tools will emerge. This platform can be used as a display window for various tools, providing relevant links to guide users in choosing which tool to use. We believe that improving and standardizing the workflow are essential to improve the quality of TCM evidence–based research, which was the starting point for this study. However, it is still difficult to directly and comprehensively define the complete and scientific workflow needed for TCM evidence–based research. We hope that this study can play a role in attracting more users to participate in this project. After using the platform, more interactive and customized functions or ideas are required to be proposed to promote continuous improvement and optimization of the platform’s functionalities.

To improve the quality of TCM evidence–based research, we should not only rely on improving the quality of systematic reviews but also lay the foundation at the level of the source data in RCT stages. When carrying out a systematic review, if we can clearly know what high-quality source data are lacking, we can also know what type of clinical study to perform next.

## Supplementary material

10.2196/49328 Multimedia Appendix 1 The technologies, tools, implementation steps, and main problems solved during the development of TCMeta (Traditional Chinese Medicine Meta-analysis).

10.2196/49328 Multimedia Appendix 2 Example operation diagram.

## Abbreviations

API: application programming interface
CCEBTCM: China Center for Evidence Based Traditional Chinese Medicine
RCT: randomized controlled trial
SMD-TCM: systematic review/meta-analysis database of traditional Chinese medicine
TCM: traditional Chinese medicine
TCMeta: Traditional Chinese Medicine Meta-analysis
