# Supplementary material for: Web-Based Platform for Systematic Reviews and Meta-Analyses of Traditional Chinese Medicine: Platform Development Study
Source: JMIR Form Res. 2024 Nov 22;8:e49328. doi: 10.2196/49328 (PMC11612523; doi:10.2196/49328)
Supplement: Multimedia Appendix 1 [file formative-v8-e49328-s001.docx]

**Multimedia Appendix 1 .** The technologies, tools, implementation steps, and main problems solved during the development of TCMeta

| **Category** | **Item** | | **Description** |
| --- | --- | --- | --- |
| **List of Typical Technologies** | Frontend | Vue.js | A progressive JavaScript framework for building user interfaces. |
|  |  | uni-app | A cross-platform Vue.js-based framework for compiling a set of code to multiple platforms such as iOS and Android. |
|  | Backend | Asp.Net 6.0  Asp.net Core WebApi | Open source developer platform launched by Microsoft for creating Web applications and services. |
|  |  | Newtonsoft Json.NET | Newtonsoft.Json (Json.NET) is .NET's most popular JSON processing library. |
|  |  | AutoMapper | A convention-based object-object mapper. |
|  | Database | MySQL | Open source relational database management system. |
|  | Other technologies | Multi-language support | Internationalization (i18n) library to support multi-language display. |
|  |  | Literature import and screening | This function has been implemented by independent programming. |
|  |  | Flowchart generation | This function has been implemented by independent programming. |
| **Development Tools** | Visual Studio Code (VSCode) | | A popular lightweight source code editor for multiple programming languages. |
|  | Visual Studio 2022 (VS2022) | | Integrated development environment (IDE), mainly for .NET application development. |
|  | MySQL Workbench | | The official graphical tool for designing and managing databases for MySQL. |
|  | HBuilderX | | A powerful IDE specifically designed for front-end development, especially suitable for uni-app development, providing smart prompts, code highlighting, and more. |
| **Implementation Step** | Requirements Analysis | | Define business objectives and determine system functional requirements. |
|  | Market and technology research | | Analyze competitive products and existing solutions to select the appropriate technology stack. |
|  | Architecture design | | Including the design of front-end and back-end separation architecture to ensure the scalability and maintainability of the system. |
|  | Database design | | According to the business logic to design a reasonable data model, and establish the corresponding database table structure. |
|  | Coding implementation | | Write front-end and back-end code according to the design scheme. |
|  | Testing | | Unit testing, integration testing, and performance testing to ensure software quality. |
|  | Deploy Release | | After the tests pass, deploy the software to production and go live. |
|  | Follow-up maintenance | | Monitor the system operation and fix the problems in time. |
| **Problem Solving** | Multilingual support | | Allows the system to adapt to both Chinese and English user groups. |
|  | Two-person screening contrast | | Provides an objective way to compare two options in order to reduce the impact of personal bias. |
|  | Standardizing the screening criteria | | Ensured that the screening process followed the same criteria for all participants, thus improving the consistency and accuracy of the screening. |
|  | Literature import | | Allows users to easily import research literature from Wanfang database and PubMed. |
|  | Automatic generation of screening flowcharts | | Automatic generation of visual flowcharts to help users understand the complex screening process. |
|  | Collation of TCM data | | Disentangle the components of prescriptions in interventions in terms of name, dosage, etc., and compare the similarities and differences of the data in detail. |
|  | Calls to third-party plugins or services | | Calls to text editing plugins, calls to PythonMeta services based on open source rules, etc. |
