# Supplementary material for: Web-Based Platform for Systematic Reviews and Meta-Analyses of Traditional Chinese Medicine: Platform Development Study
Source: JMIR Form Res. 2024 Nov 22;8:e49328. doi: 10.2196/49328 (PMC11612523; doi:10.2196/49328)
Supplement: Multimedia Appendix 2 [file formative-v8-e49328-s002.docx]

Example operation diagram


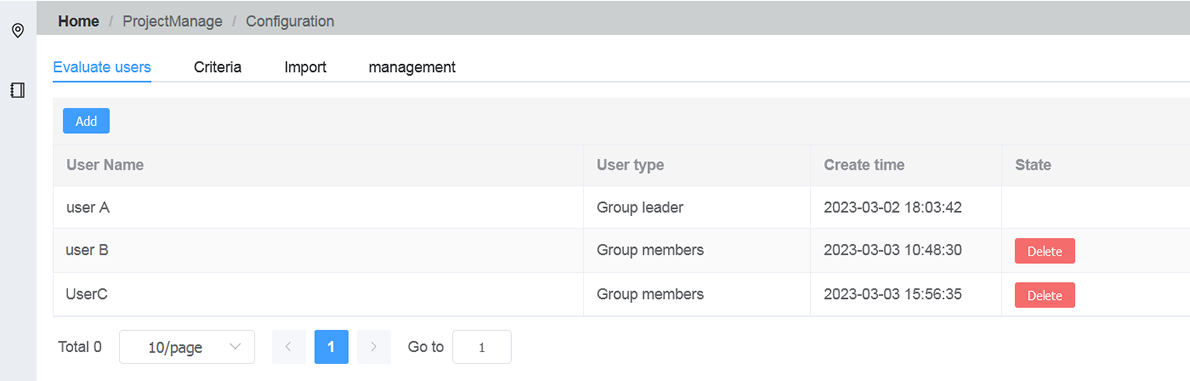


Organizing research groups.


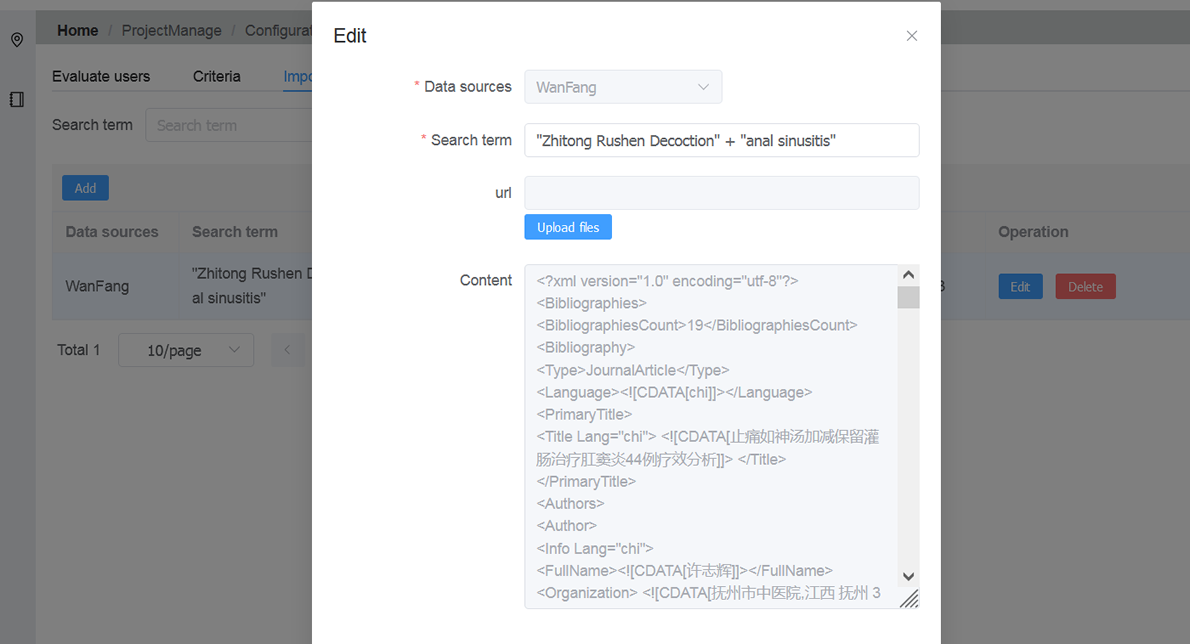


The literature data were imported from WanFang.


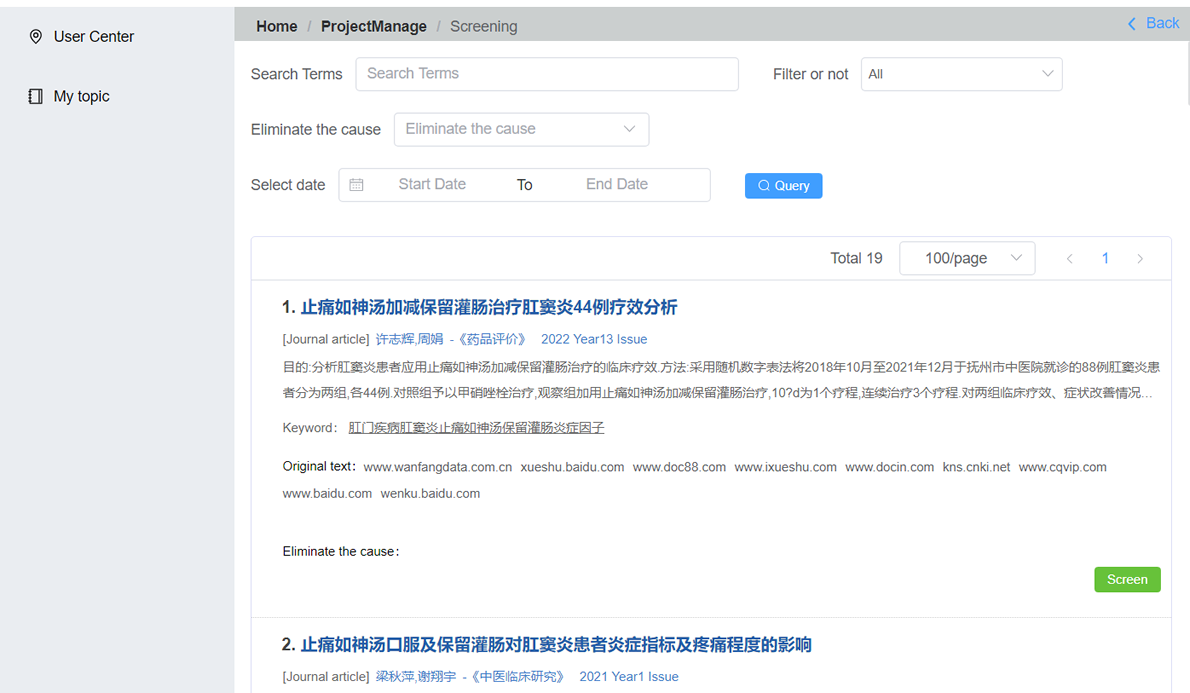


Click on "Original text" to read the original text online.


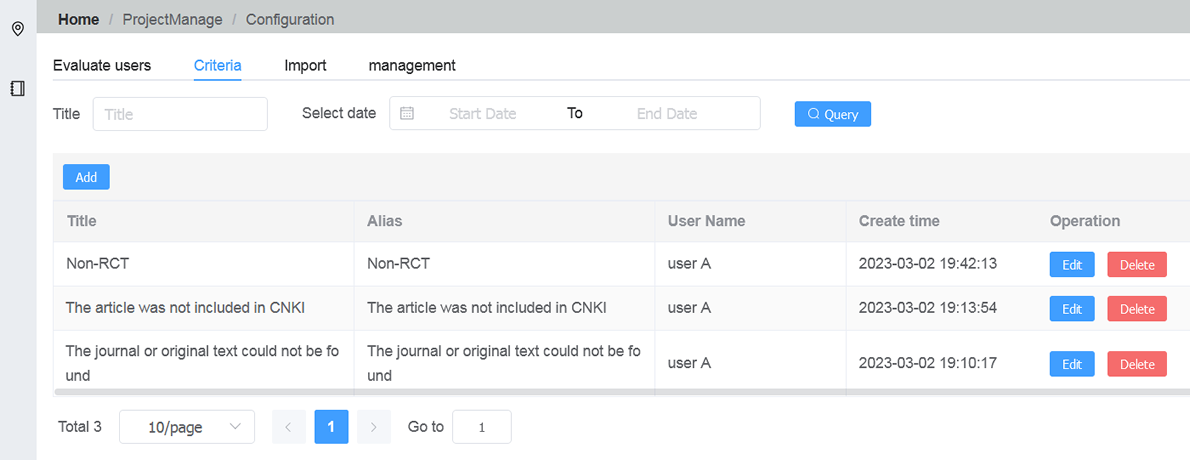


Add literature screening rules.


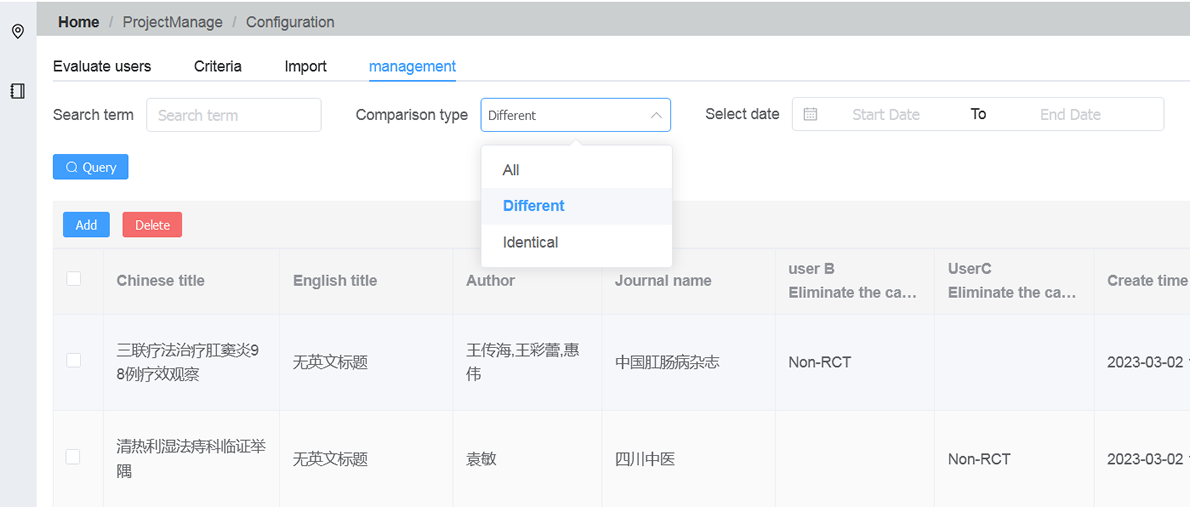


The differences in literature screening between the two members were compared.


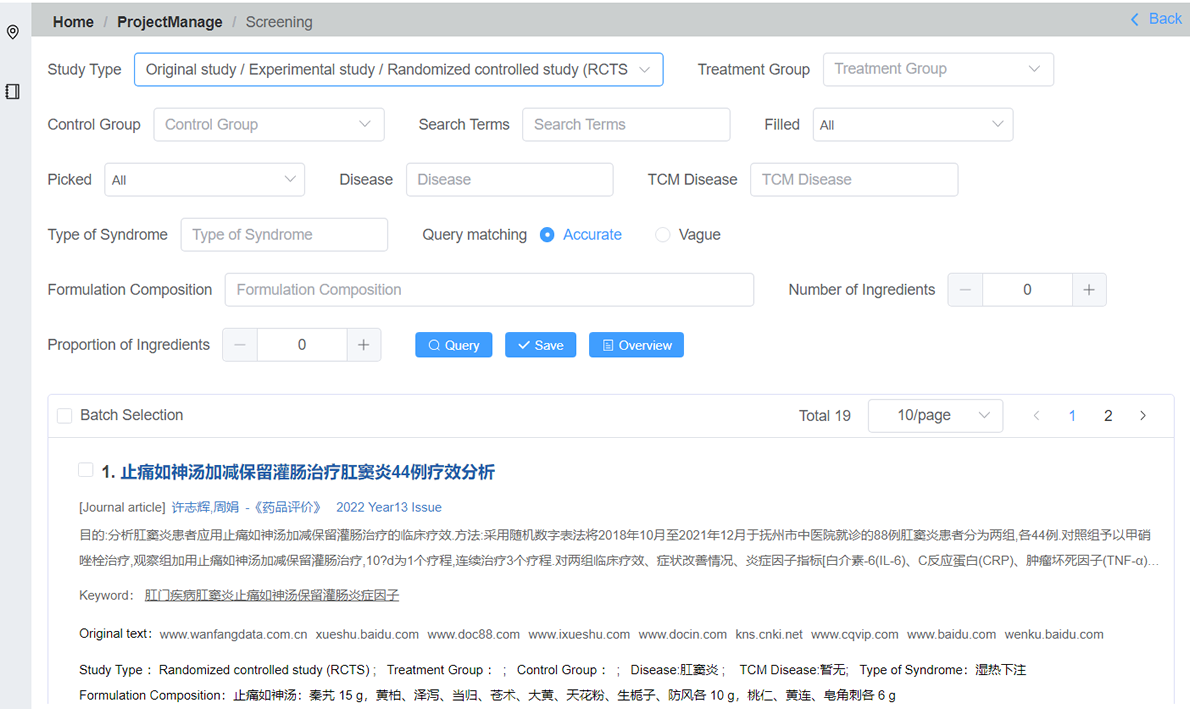


The primary interface for query mode.


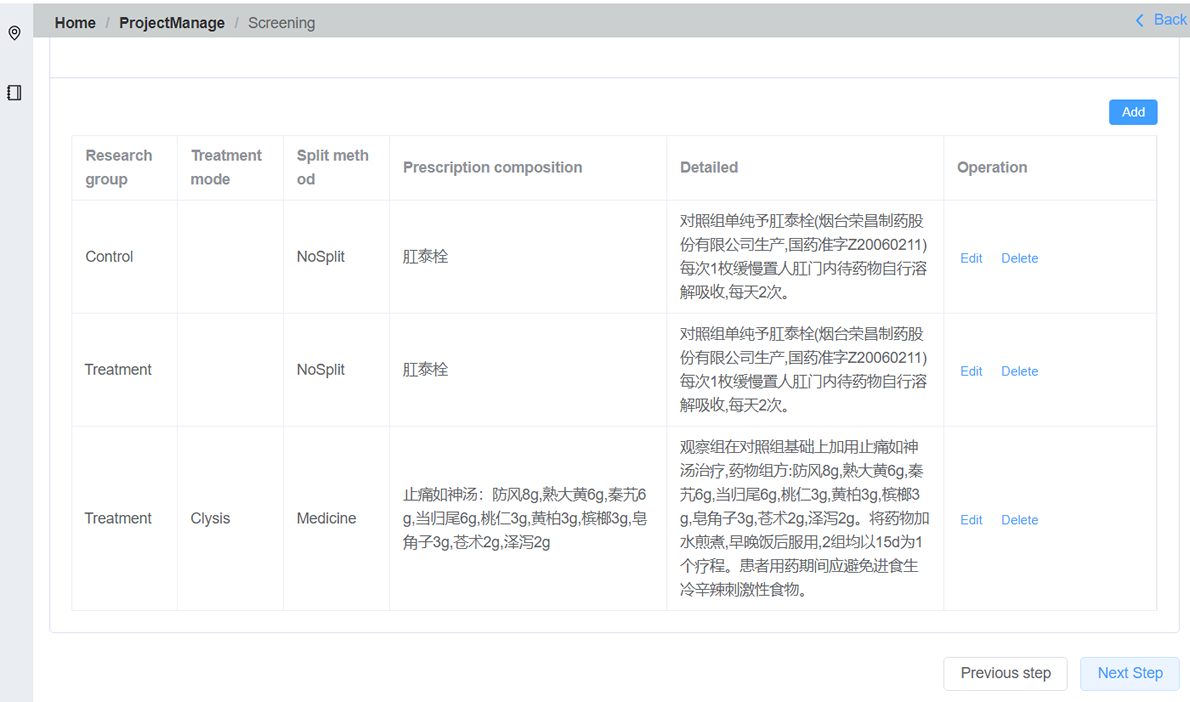


The interventions for each group were listed.


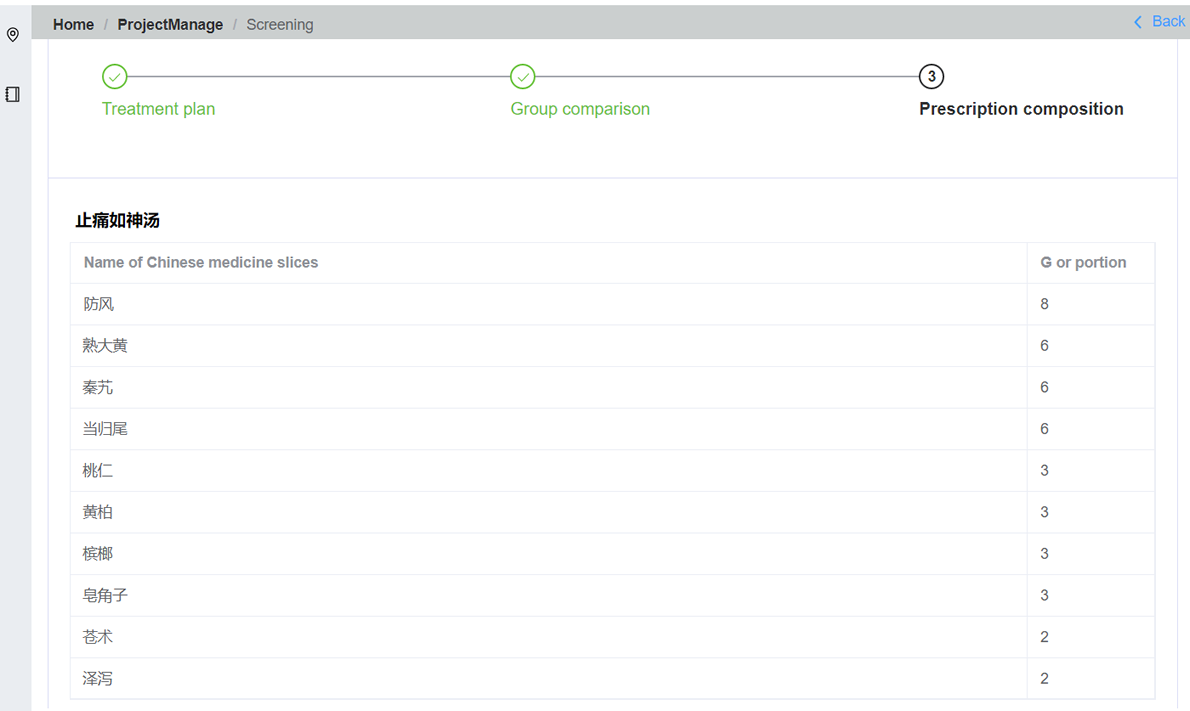


List the herbs and weights in the TCM prescription.


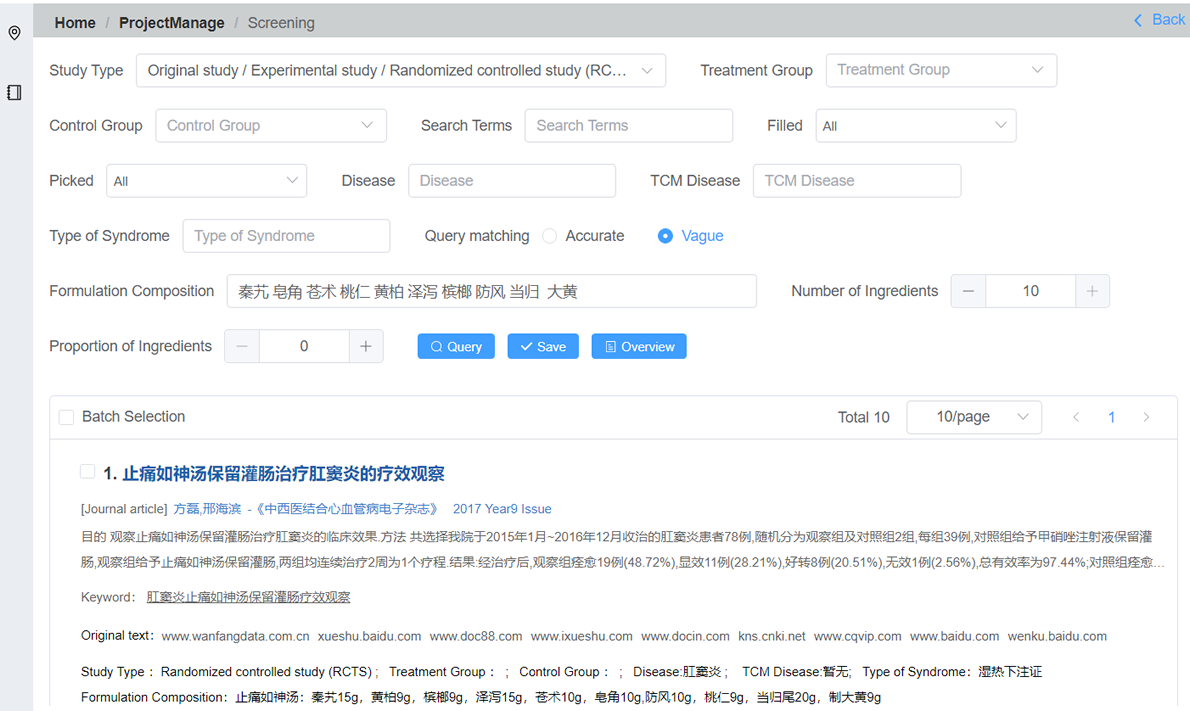


Use Query matching, Number of Ingredients, Proportion to Ingredients and other options to find partially or exactly the same prescription.


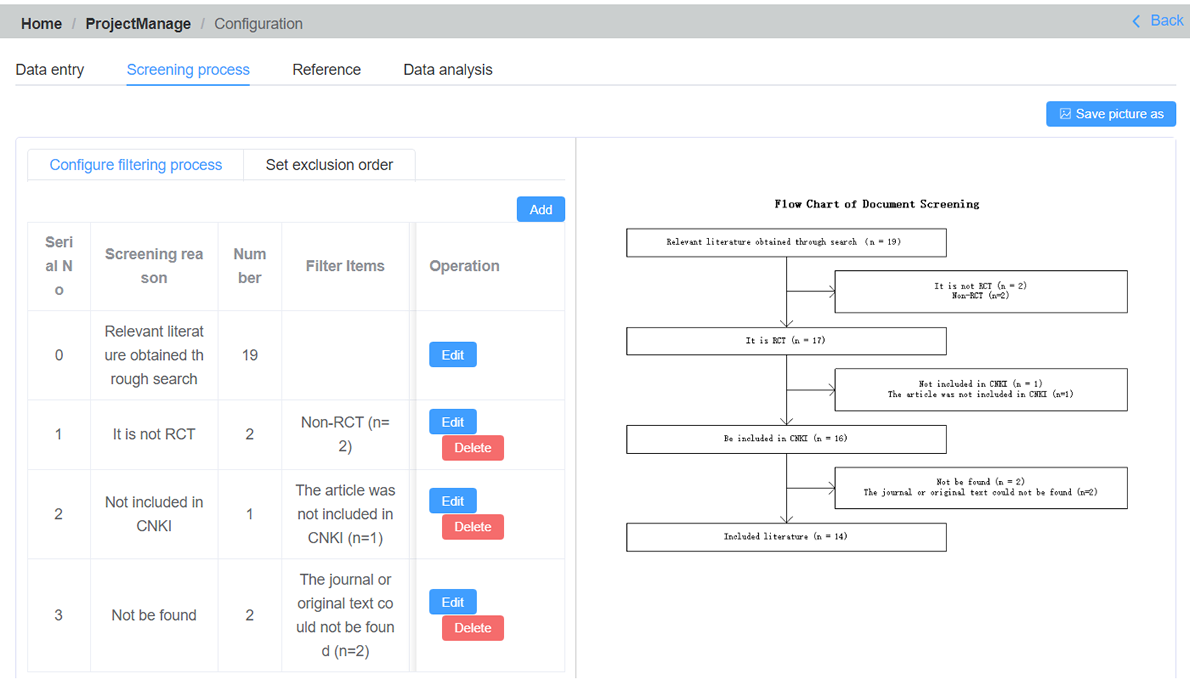


Flow Chart.


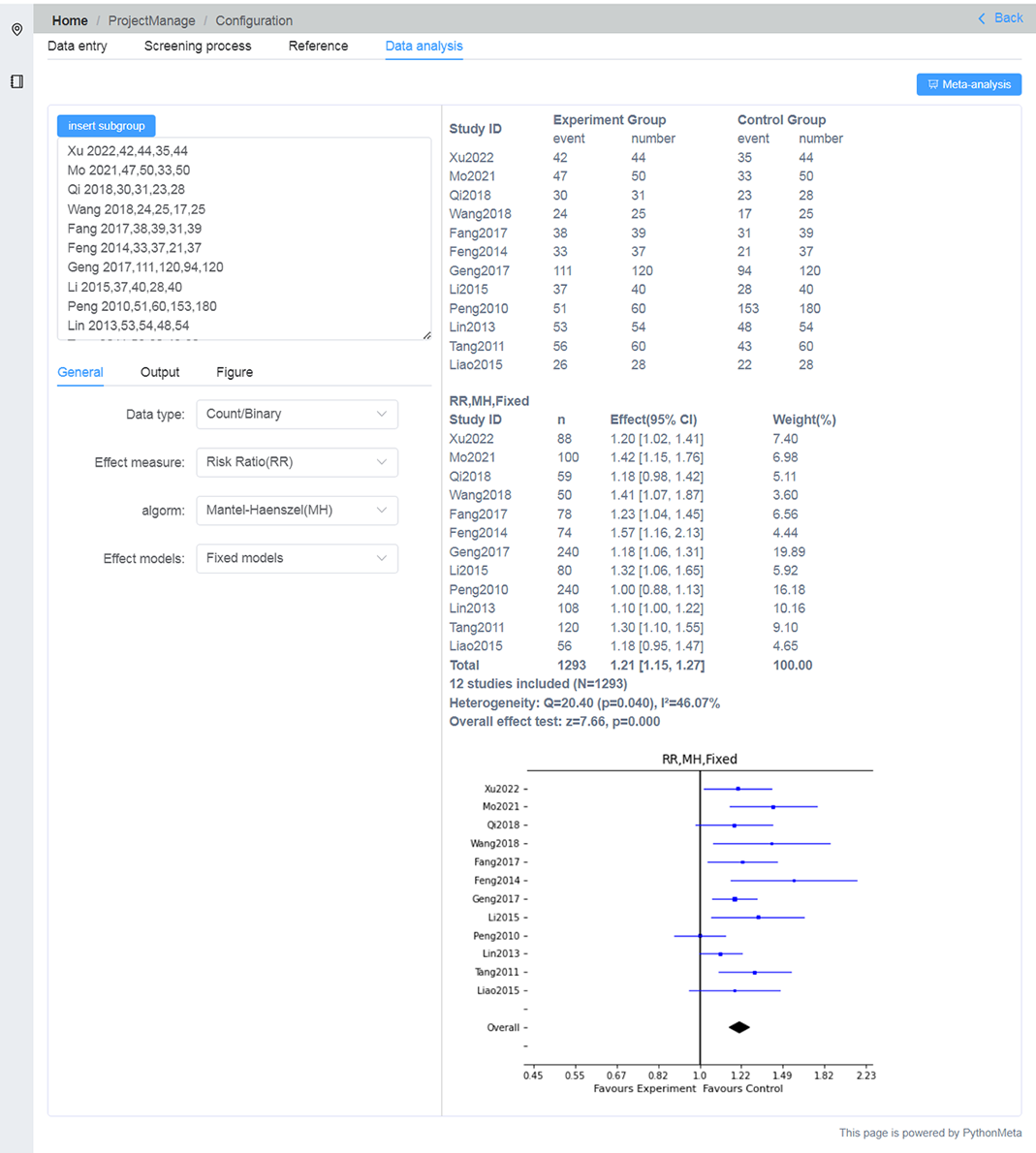


The forest plot was generated online via PythonMeta.
